# 1788. High Ferritin Levels in Hospitalized Patients with Acute Babesiosis in a Tertiary Medical Center in Long Island, NY (2014-2022)

**DOI:** 10.1093/ofid/ofad500.1617

**Published:** 2023-11-27

**Authors:** Luis A Marcos, Evan Gary, Pooja Lamba, Miguel A Saldivar, Andreas Kalogeropoulos, Eric Spitzer

**Affiliations:** Stony Brook University Hospital, Stony Brook, New York; STONY BROOK UNIVERSITY, Syosset, New York; Stony Brook University, Stony Brook, New York; SUNY Stony Brook University Hospital, Stony Brook, NY; Stony Brook University, Stony Brook, New York; Stony Brook Medicine, Stony Brook, New York

## Abstract

**Background:**

Babesiosis, an intra-erythrocytic protozoan disease caused by *Babesia spp*, is an emerging zoonotic parasitic disease worldwide. Our group have recently described the utility of biomarkers such as lipid profile and procalcitonin levels in acute babesiosis caused by *B. microti*. Ferritin is a nonspecific marker, but serum levels can be seen with severe infections including bacterial and viral processes. Anecdotal reports suggest that elevated ferritin levels were elevated in a series of cases of acute babesiosis. Our aim is to investigate any association between ferritin levels and severity of infection and other biomarkers in patients diagnosed with acute babesiosis in Long Island, New York.

Correlation between Ferritin and LDH levels in hospitalized patients with acute babesiosis (r=0.565, P=0.0004).

**Figure:**
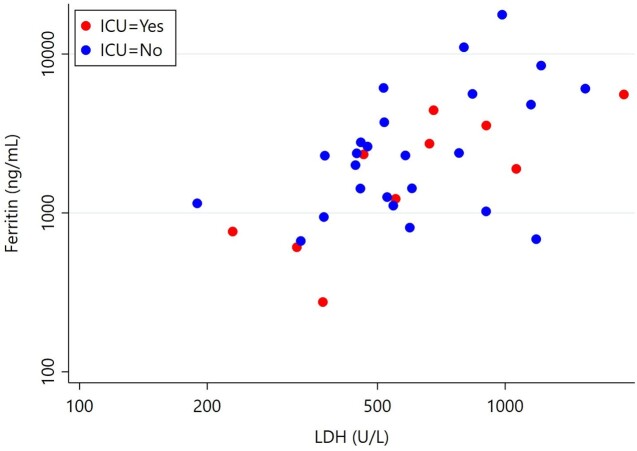

**Methods:**

We conducted a retrospective chart review of adult patients who had a positive peripheral blood thin smear for intra-erythrocyte *B. microti* parasites at Stony Brook University and Southampton Hospitals (Stony Brook Medicine) between 2014 and 2022. All cases were confirmed by PCR analysis which was performed by the NY State Department of Health. Only patients who had a ferritin level drawn during acute presentation were included in the analysis.

**Results:**

A total of 36 patients (n=6, 16.6% female) met criteria for acute *Babesia* infection and had ferritin level drawn on admission during 2014-2022. Average age was 64 years-old (range: 42–90). Four patients (11.1%) were asplenic and 10 (27.7%) required ICU level of care. Parasitemia median was 1.6% (range: 0.1%-19.5%), eight cases (22.2%) required RBC exchange transfusion. Ferritin level median was 2297 ng/mL (range: 275-17,684). All cases had ferritin levels above 600ng/mL except one. Ferritin levels were higher on the group of patients who were not admitted to ICU (median: 2301ng/mL) than those admitted to ICU (median: 1894ng/mL) (p=0.25). In linear regression analysis, there was a correlation between ferritin and lactate dehydrogenase (LDH) levels (r=0.565, P=0.0004; Fig 1).

**Conclusion:**

Ferritin was found strongly correlated with LDH levels. However, clinical severity of babesiosis is difficult to predict from inflammatory markers since low levels of ferritin and LDH were seen in ICU patients (Fig. 1). High ferritin levels were seen in almost all patients on this study.

**Disclosures:**

**All Authors**: No reported disclosures

